# Ameliorative Effects of some Natural Antioxidants against Blood and Cardiovascular Toxicity of Oral Subchronic Exposure to Silicon Dioxide, Aluminum Oxide, or Zinc Oxide Nanoparticles in Wistar Rats

**DOI:** 10.1155/2023/8373406

**Published:** 2023-03-11

**Authors:** Riyadh Musaed Naji, Mohamed A. Bashandy, Abdallah H. Fathy

**Affiliations:** ^1^Department of Zoology, Faculty of Science, Al-Azhar University, Cairo 11651, Egypt; ^2^Department of Zoology, Faculty of Science and Education, Aden University, Yemen; ^3^Department of Animal House Facility, Faculty of Pharmacy, Ain Shams University, Cairo 11566, Egypt

## Abstract

The present study determines the possible protective role of fig fruit extract with olive oil and date palm fruit extract (FOD) in decreasing the oral subchronic blood and cardiovascular toxicity of SiO_2_NPs, Al_2_O_3_NPs, or ZnONPs. The present study used 80 male Wistar rats (8 groups, *n* = 10) distributed according to the treatment. The FOD treatments were used at their recommended antioxidant doses. All nanoparticles (NPs) were given orally and daily at doses of 100 mg/kg for 75 days. The oral administration of different NPs alone led to dramatic, oxidative stress, inflammatory markers, blood coagulation, endothelial dysfunction markers, myocardial enzymes, hematological parameters, lipid profile, and histopathological features compared with the control group. The FOD-NP-treated groups recorded significantly ameliorated blood and cardiovascular toxicity hazards compared to the groups administered with the NPs alone. In conclusion, the administration of FOD provides considerable chemopreventive and ameliorative effects against NP toxicity.

## 1. Introduction

Human exposure to nanomaterials is increased with the increased application of nanotechnology [1]. During the nanoparticle's transport in the blood, it can alter the blood and the blood vessels [2]. However, nanoparticles can be translocated between all organs causing toxicity at the whole-body level [3, 4].

Silica nanoparticles, also known as silicon dioxide (SiO_2_NPs), have good property including large surface-area-to-volume ratios, biocompatibility, and ease of modification, so it is considered a good tool in biomedicine and biotechnology [5, 6]. Studies on silica nanoparticles indicated that exposure to these nanoparticles and their accumulation in various organs could induce ROS generation, cause oxidative stress, proinflammatory stimulation, many side effects, and toxicity [7, 8]. Aluminum oxide nanoparticles (Al_2_O_3_NPs), a class of porous nanomaterials, belong to the family of metal oxide nanomaterials and contribute to 20% of all nanosized chemicals. In addition, the bioinertness and easy surface functionalization allow their use in the biological environment [9]. Following the EU and US EPA criteria, the toxicity of Al_2_O_3_NPs was rated high [10]. Al_2_O_3_NPs have been listed as one of the metal oxide nanoparticles that are harmful to organisms [11]. Zn is an essential element with fundamental biological functions. The superior properties render ZnONPs used in different applications and biomedical imaging [12, 13]. Exposure to zinc oxide NPs might lead to toxicity to various body organs [14].

The body has a built-in defense mechanism to protect itself from free radical damage but eventually aging and diseases deplete the body of antioxidants [15]. It was thought that dietary antioxidants act as radical scavengers and decrease free radical attacks on cellular molecules [16].

The olive tree was considered one of the oldest trees. The olive oil phenolic compounds were found to possess antimicrobial, antioxidant, inhibitors of LDL-C oxidation and anti-inflammatory activities as well as affect the early phases of atherosclerosis [17]. Boskou [18] reported that olive oil is abundant in linoleic acid (3% to 21%) and oleic acid (56% to 84%) of the oil.

The dried fig fruit was found to contain alkaloids, flavonoids, coumarins, saponins, sterols, and terpenes that show therapeutic and anti-inflammatory activities and promote apoptosis [19, 20].

The date palm is one of the oldest known fruit crops. In addition, date palm fruit contains thirteen flavonoid glycosides that possess antineoplastic effects such as quercetin and glucans [21–23]. Studies on the extract of date palm fruit confirmed that it possesses antioxidative, antimutagenic, antimicrobial, and antimutagenic activities. In addition, date palm fruit is also traditionally used to treat many diseases like hypertension [24].

The present work aims at investigating the possible chemoprevention of FOD against different nanoparticle-induced subchronic blood and cardiovascular toxicity in Wistar rats including oxidative stress, inflammatory markers, blood coagulation, endothelial dysfunction markers, and myocardial enzymes, hematological parameters, lipid profile, and histopathological features.

## 2. Materials and Methods

### 2.1. Chemicals

We used high-grade chemicals.

### 2.2. Nanoparticles

**Figure 1:**
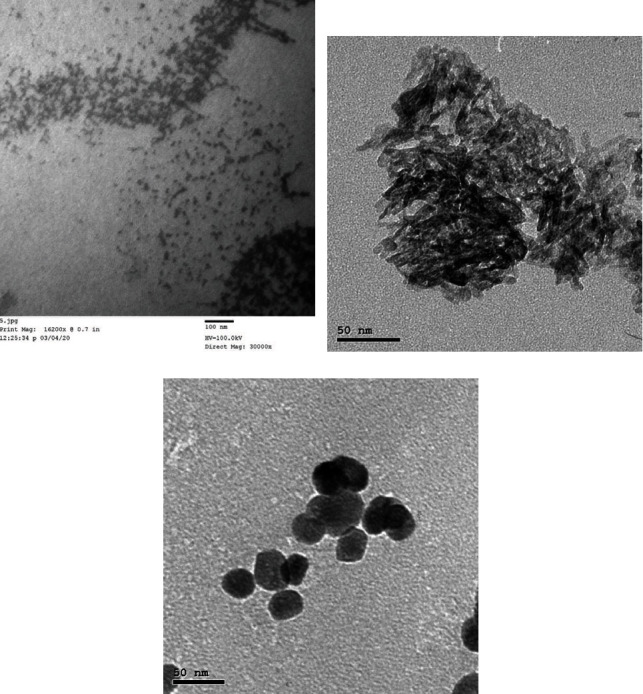
Transmission electron microscope (TEM) images of silica nanoparticles (SiO2NPs) (a), alumina nanoparticles (Al_2_O_3_NPs) (b), and zinc oxide nanoparticles (ZnONPs) (c).

The SiO_2_NPs, Al_2_O_3_NPs, or ZnONPs were prepared at an average size of less than 50 nm and characterized in the Nanogate Laboratory, Cairo, Egypt. The structure of the nanoparticle was confirmed using a TEM (JEM-2100, Jeol, Akishima, Japan) at a voltage of 200 kV and X-ray diffraction (XRD) analysis using a powder diffractometer system (X'pertPro-Panalytical, Malvern, United Kingdom) as shown in Figure 1.

### 2.3. Preparation of Nanoparticle (NP) Treatments

The different nanoparticles were homogeneously suspended in water and vibrated by vortex before administration to rats. All nanoparticles were given orally (100 mg/kg.b.wt) for 75 days. The used doses were confirmed by a pilot study conducted in our lab (data not shown). The doses of SiO_2_NPs were following Gmoshinski et al. [25], while Al_2_O_3_NP doses were following Park et al. [26], and the doses of ZnONPs were following Yousef et al. [27].

### 2.4. Plant Materials and Authorities

We purchased the extravirgin olive oil from the Grup Pons Company (Spain), the fig fruit from Kafoods Ltd. (Turkey), and the date palm fruit from the Al-MADINA AL-MUBARAK market (Saudi Arabia). The plant materials were identified and authenticated by Dr. Al-Baraa El-Saied, Al-Azhar University, Egypt. The voucher specimens were placed in the unit of medicinal plants.

### 2.5. Preparation of Crude Extracts

The fig fruit hydroalcoholic extract was prepared according to the method of Gilani et al. [28], while the hydroalcoholic extract of the date fruit was prepared according to the method of Al-Qarawi et al. [29].

### 2.6. Preparation of the Antioxidant Treatments

The extravirgin olive oil (7 g/kg.b wt.) and the fig and date palm fruit extracts (1 g/kg.b wt) were supplemented on rats orally and daily by gavage [30]. The different human-recommended antioxidant doses of the used materials were calculated and converted to rat doses [31].

### 2.7. The Experimental Animals

We purchased the male Wistar rats from VACSERA, Giza, Egypt. The study was conducted at the animal house, Faculty of Pharmacy, Ain Shams University, Cairo, Egypt, under standard conditions of a temperature-controlled environment, food, and water *ad libitum*.

### 2.8. Animal Welfare

The animal experiment was conducted under the National Research Centre guidelines [32], and the study was approved by an independent ethics committee of the Faculty of Pharmacy, Ain Shams University.

### 2.9. Experimental Design

The present study used 80 male Wistar rats of average weights from 150 g to 170 g and divided into 8 groups (*n* = 10) as the following:

*Group I (control)*: this group did not receive any treatments for 75 days.

*Group II (FOD)*: this group was treated orally and daily with the antioxidants (extravirgin olive oil (7 g/kg), fig extract (1 g/kg), and date palm fruit extract (1 g/kg)) for 2 weeks before and 75 days during the experiment.

*Group III (SiO_2_NPs)*: this group was administered orally and daily with silicon oxide nanoparticles (SiO_2_NPs, 100 mg/kg.bwt).

*Group IV (FOD-SiO_2_NPs)*: this group was treated with FOD and SiO_2_NPs on the same schedule mentioned above in groups II and III.

*Group V (Al_2_O_3_NPs)*: this group was administered orally and daily with aluminum oxide nanoparticles (Al_2_O_3_NPs, 100 mg/kg.bwt).

*Group VI (FOD-Al_2_O_3_NPs)*: this group was treated with FOD and Al_2_O_3_NPs on the same schedule mentioned above in groups II and V.

*Group VII (ZnONPs)*: this group was administered orally and daily with aluminum oxide nanoparticles (ZnONPs, 100 mg/kg.bwt).

*Group VIII (FOD-ZnONPs)*: this group was treated with FOD and ZnONPs on the same schedule mentioned above in groups II and VII.

### 2.10. Collection and Preparation of Samples

The drained blood samples from each animal under anesthesia were divided into two portions, one portion was put in plain tubes for serum preparation (centrifuged at 1006 g for 10 min), and the other portion was collected in a vial containing 0.5 M EDTA for hematological measurements. The separated sera samples were frozen at -80°C until future use. After that, the hearts were immediately dissected and fixed in 10% neutral buffered formalin for histopathological preparations.

### 2.11. Biochemical Study

#### 2.11.1. Oxidative Stress Markers

We used ready-made kits from the Bio-diagnostic Co., Egypt in the measurements of the serum systemic oxidative stress markers (reduced glutathione (GSH), superoxide dismutase (SOD), total antioxidant capacity (TAC), and thiobarbituric acid reactive substances (TBARS)).

The reactive oxygen species modulator-1 (ROS) was estimated in the serum of rats by ELISA technique according to the instructions provided with the kit. The used kit was purchased from MyBioSource, Inc. San Diego, CA 92195-3308, USA (Catalog No: MBS775259).

#### 2.11.2. Inflammatory Markers

The interleukin-1beta (IL-1*β*) (Elabscience Biotechnology, Inc., Texas, USA, Catalog No: E-EL-R0012), the interleukin-6 (IL-6) (Elabscience Biotechnology, Inc., Texas, USA, Catalog No: E-EL-R0015), and the tumor necrosis (TNF-*α*) (MyBioSource, Inc. San Diego, CA 92195-3308, USA, Catalog No: MBS2507393) were estimated in the serum of the rats by ELISA technique using methods outlined in the kits.

#### 2.11.3. Blood Coagulation and Endothelial Dysfunction Markers

The D-Dimer (Catalog No: MBS700162, MyBioSource, Inc. San Diego, CA 92195-3308: USA), the endothelin-1 (ET-1) (Catalog No: MBS704215, MyBioSource, Inc. San Diego, CA 92195-3308, USA), the P-selectin (Catalog No: MBS727217, MyBioSource, Inc. San Diego, CA 92195-3308, USA), intercellular adhesion molecule-1 (ICAM-1/CD54) (Catalog No: E-EL-R285096T, Elabscience Biotechnology, Inc., Texas, USA), and the vascular cell adhesion molecule-1 (VCAM-1) (Catalog No: E-EL-R1061, Elabscience Biotechnology, Inc., Texas, USA) were estimated in the blood serum of the rats by ELISA technique using methods outlined in the diagnostic kits.

#### 2.11.4. The Measurements of Serum Myocardial Enzymes

The lactate dehydrogenase (LDH) was measured in the serum following the method of Mittal et al. [33] using commercial kits purchased from Abnova Company USA. The creatine phosphokinase (CPK) was measured in the serum following the method of Steen et al. [34] using commercial kits purchased from Abnova Company, USA. The creatine kinase MB isoenzyme (CK-MB) (Catalog No: E-EL-R1327, Elabscience Biotechnology, Inc., Texas, USA) was estimated in the blood serum of the rats by ELISA technique using methods outlined in the diagnostic kits.

The CK index was calculated to confirm the heart damage as the following:

(1) $$\begin{array}{c} \text{The CK index}=\left(\text{CK}-\text{MB}\left(\text{ng}/\text{mL}\right)\right)\times 100/\left(\text{total CK activity}\left(\text{IU}/\text{L}\right)\right). \end{array}$$

#### 2.11.5. Measurements of Hematological Parameters and Blood Indices

The hematocrit (Hct), red blood corpuscle (RBC), hemoglobin (Hb), Platelet count, white blood cell (WBC) count, differential leukocyte count, and blood indices) were estimated in the blood samples containing 0.5 M EDTA by the autoanalyzer (CBC counter, Sino thinker, sk9000, U.S.A).

#### 2.11.6. The Measurements of Lipid Parameters and their Risks

The triglycerides (TG), total cholesterol (TC), low-density lipoprotein cholesterol (LDL-C), high-density lipoprotein cholesterol (HDL-C), and nonhigh-density lipoprotein cholesterol (non-HDL-C), and risk ratios were estimated in the blood serum using readymade kits from Bio-diagnostic Co., Egypt, for research kits.

#### 2.11.7. The Histopathological Study

The heart tissue samples were dissected from each animal, fixed, processed in alcohols, sectioned (5 *μ*m), and stained with Hx & E according to certain methods [35].

### 2.12. Statistical Analysis

We analyze the data by using SPSS/PC program. The results were expressed as mean ± SE. The data were analyzed using one-way ANOVA followed by LSD post hoc for comparisons (*p* < 0.05).

## 3. Results

### 3.1. The Effects of FOD on the Serum Oxidative Stress Markers of Rats Administered with SiO_2_NPs, Al_2_O_3_NPs, or ZnONPs for 75 Days Are Represented in Table 1

The FOD-treated group shows insignificant changes in TAC, GSH, ROS, SOD, and TBARS in the serum when compared with the control group.

The SiO_2_NPs, Al_2_O_3_NPs, and ZnONP-treated groups recorded a significant reduction in the TAC (36.84%, 52.51%, and 58.45%, respectively), GSH (9.89%, 13.51%, and 16.57%, respectively), and SOD (13.98%, 24.17%, and 30.37%, respectively) in the serum, in contrast to a significant elevation in the ROS (107.13%, 257.93%, and 325.06%, respectively), and TBARS (22.87%, 46.39%, and 64.86%, respectively) in the serum as compared with the control group.

In the same concern, the FOD-SiO_2_NPs, FOD-Al_2_O_3_NPs, and FOD-ZnONP-treated groups recorded a significant reduction in the TAC (21.50%, 37.33%, and 40.43%, respectively), GSH (4.38%, 5.17%, and 9.28%, respectively), and SOD (7.58%, 17.36%, and 19.08%, respectively), in the serum in contrast to a significant elevation in the ROS (82.07%, 219.08%, and 277.47%, respectively), and TBARS (11.46%, 32.31%, and 42.35%, respectively) in the serum as compared with the control group.

The FOD-SiO_2_NPs, FOD-Al_2_O_3_NPs, and FOD-ZnONP-treated groups recorded a significant elevation in the TAC, GSH, and SOD in the serum, in contrast to a significant reduction in the ROS, and TBARS in the serum as compared with the SiO_2_NPs, Al_2_O_3_NPs, and ZnONP-treated groups, respectively.

### 3.2. The Effects of FOD on the Serum Inflammatory Markers of Rats Administered with SiO_2_NPs, Al_2_O_3_NPs, or ZnONPs for 75 Days Are Represented in Figures 2–4

The FOD-treated group showed insignificant changes in the serum TNF-*α*, serum IL-I*β*, and serum IL-6 when compared with their corresponding control values.

The SiO_2_NPs, Al_2_O_3_NPs, and ZnONP-treated groups recorded a significant elevation in the TNF-*α* (34.23%, 42.70%, and 54.95%, respectively), IL-I*β* (273.12%, 432.48%, and 748.32%, respectively), and IL-6 (35.95%, 46.58%, and 74.24%, respectively) in the serum as compared with the control group.

In the same concern, the FOD-SiO_2_NPs, FOD-Al_2_O_3_NPs, and FOD-ZnONP-treated groups recorded a significant elevation in the TNF-*α* (23.42%, 23.06%, and 29.37%, respectively), IL-I*β* (169.60%, 345.28%, and 595.52%, respectively), and IL-6 (22.71%, 30.42%, and 34.79%, respectively) in the serum when compared with the control.

Moreover, the FOD-SiO_2_NPs, FOD-Al_2_O_3_NPs, and FOD-ZnONP-treated groups recorded a significant reduction in the serum TNF-*α*, serum IL-I*β*, and serum IL-6 when compared with their corresponding values in the SiO_2_NPs, Al_2_O_3_NPs, and ZnONP-treated groups, respectively.

### 3.3. The Effects of FOD on the Blood Coagulation and Endothelial Dysfunction Markers Serum Inflammatory Markers of Rats Administered with SiO_2_NPs, Al_2_O_3_NPs, or ZnONPs for 75 Days Are Represented in Table 2

The FOD-treated group shows insignificant changes in the serum D-dimer, serum ET-1, serum P-selectin, serum ICAM-1, and serum VCAM-1 when compared with their corresponding control values.

The SiO_2_NPs, Al_2_O_3_NPs, and ZnONP-treated groups recorded a significant elevation in the D-dimer (143.23%, 182.35%, and 290.27%, respectively), ET-1 (69.65%, 157.17%, and 268.40%, respectively), P-selectin (96.56%, 91.31%, and 124.73%, respectively), ICAM-1 (91.63%, 122.59%, and 155.61%, respectively), and VCAM-1 (118.83%, 142.97%, and 172.89%, respectively) in the serum as compared with the control.

In the same concern, the FOD-SiO_2_NPs, FOD-Al_2_O_3_NPs, and FOD-ZnONP-treated groups recorded a significant elevation in the D-dimer (93.34%, 134.78%, and 234.78%, respectively), ET-1 (34.51%, 123.70%, and 217.46%, respectively), P-selectin (46.58%, 52.44%, and 86.84%, respectively), ICAM-1 (57.81%, 98.04%, and 130.54%, respectively), and VCAM-1 (92.10%, 121.95%, and 142.65%, respectively) in the serum as compared with the control.

In addition, the FOD-SiO_2_NPs, FOD-Al_2_O_3_NPs, and FOD-ZnONP-treated groups recorded a significant reduction in the serum D-dimer, serum ET-1, and serum P-selectin, serum ICAM-1, and serum VCAM-1 as compared with their corresponding values in the SiO_2_NPs, Al_2_O_3_NPs, and ZnONP-treated groups, respectively.

### 3.4. The Effects of FOD on the Serum Myocardial Enzymes of Rats Administered with SiO_2_NPs, Al_2_O_3_NPs, or ZnONPs for 75 Days Are Represented in Table 3

The FOD-treated group showed insignificant changes in the LDH, CPK, CK-MB, and CK index in the serum as compared with the control.

In addition, the SiO_2_NPs, Al_2_O_3_NPs, and ZnONP-treated groups recorded a significant elevation in the LDH (53.41%, 86.01%, and 87.33%, respectively), CPK (22.43%, 36.90%, and 41.62%, respectively), CK-MB (1793%, 3736%, and 3194%, respectively), and CK index (1452%, 2685%, and 2811%, respectively) in the serum as compared with the control.

Similarly, the FOD-Al_2_O_3_NPs and FOD-ZnONP-treated groups recorded a significant elevation in the LDH (55.01%, and 60.51%, respectively), CPK (25.50%, and 28.40%, respectively), CK-MB (2830%, and 3194%, respectively), CK index (2230%, and 2455%, respectively) in the serum as compared with the control.

In the same concern, the FOD-SiO_2_NP-administered group showed a significant elevation in the LDH (29.82%), CPK (10.03%), CK-MB (1265%), and CK index (1141%) in the serum when compared with the control.

Moreover, the FOD-SiO_2_NPs, FOD-Al_2_O_3_NPs, and FOD-ZnONP-treated groups recorded a significant reduction in the LDH, CPK, CK-MB, and CK index in the serum as compared with the SiO_2_NPs, Al_2_O_3_NPs, and ZnONP-treated groups, respectively.

### 3.5. The Effects of FOD on the Hematological Parameters and Blood Indices of Rats Administered with SiO_2_NPs, Al_2_O_3_NPs, or ZnONPs for 75 Days Are Represented in Table 4

The FOD-treated group shows insignificant changes in the Hct, RBC count, Hb, MCV, MCH, MCHC, platelet count, WBC count, neutrophils percentage, lymphocytes percentage, and monocytes percentage in the blood when compared to the control group.

The SiO_2_NPs, Al_2_O_3_NPs, and ZnONP-treated groups recorded a significant reduction in the Hct (19.89%, 32.18%, and 38.01%, respectively), RBC count (11.16%, 22.68%, and 27.57%, respectively), Hb (36.59%, 44.38%, and 49.07%, respectively), MCV (9.59%, 12.03%, and 14.34%, respectively), MCH (28.33%, 27.79%, and 29.78%, respectively), MCHC (20.63%, 16.20%, and 17.68%, respectively), platelet count (25.43%, 33.22%, and 31.75%, respectively) in contrast to a significant elevation in the WBC count (33.60%, 43.70%, and 37.64%, respectively), neutrophils percentage (55.35%, 61.89%, and 74.53%, respectively), lymphocytes percentage (23.18%, 32.96%, and 44.93%, respectively), and monocytes percentage (51.05%, 61.28%, and 63.32%, respectively) in the blood when compared with the control group.

Similarly, the FOD-SiO_2_NPs, FOD-Al_2_O_3_NPs, and FOD-ZnONP-treated groups recorded a significant reduction in the Hct (13.85%, 24.36%, and 32.64%, respectively), RBC count (3.99%, 10.90%, and 18.78%, respectively), Hb (24.80%, 36.24%, and 40.21%, respectively), MCV (10.07%, 14.84%, and 17.28%, respectively), MCH (21.33%, 28.07%, and 26.69%, respectively), MCHC (12.68%, 15.32%, and 10.85%, respectively), platelet count (13.66%, 15.84%, and 19.39%, respectively) in contrast to a significant elevation in the WBC count (19.39%, 22.43%, and 20.92%, respectively), neutrophils percentage (26.35%, 32.57%, and 31.01%, respectively), lymphocytes percentage (12.65%, 12.27%, and 14.15%, respectively), and monocytes percentage (26.63%, 30.12%, and 32.66%, respectively) in the blood as compared with the control.

In addition, the FOD-SiO_2_NPs, FOD-Al_2_O_3_NPs, and FOD-ZnONP-treated groups recorded a significant elevation in the Hct percentage, RBC count, Hb conc., and platelet count, in contrast to a significant reduction in the WBC count, neutrophils percentage, lymphocytes percentage, and monocytes percentage in the blood as compared with the SiO_2_NPs, Al_2_O_3_NPs, and ZnONP-treated groups, respectively.

In addition, the FOD-SiO_2_NPs, FOD-Al_2_O_3_NPs, and FOD-ZnONP-administered groups did not record any changes in the MCV, MCH, and MCHC in the blood as compared with the SiO_2_NPs, Al_2_O_3_NPs, and ZnONP-administered groups, respectively.

### 3.6. The Effects of FOD on the Serum Lipid Parameters and Their Risks of Rats Administered with SiO_2_NPs, Al_2_O_3_NPs, or ZnONPs for 75 Days Are Represented in Table 5

The FOD and the FOD-SiO_2_NP-treated groups did not show any changes in the serum lipid parameters and their risks when compared with the control.

The SiO_2_NPs, Al_2_O_3_NPs, and ZnONP-treated groups recorded a significant elevation in the TG (19.37%, 48.95%, and 54.97%, respectively), TC (16.41%, 38.22%, and 43.05%, respectively), LDL-C (20.78%, 35.93%, and 51.52%, respectively), non-HDL-C (36.41%, 82.19%, and 93.50%, respectively), TG/HDL-C (19.37%, 48.95%, and 54.97%, respectively), TC/HDL-C (16.41%, 38.22%, and 43.05%, respectively), and LDL-C/HDL-C (20.78%, 35.93%, and 51.52%, respectively), in contrast to a significant reduction in the HDL-C (17.84%, 37.04%, and 43.32%, respectively) in the serum as compared with the control.

In the same concern, the FOD-Al_2_O_3_NPs and FOD-ZnONP-treated groups recorded a significant elevation in the TG (27.23%, and 35.08%, respectively), TC (21.62%, and 26.45%, respectively), LDL-C (19.48%, and 20.78%, respectively), non-HDL-C (48.01%, and 59.33%, respectively), TG/HDL-C (27.23%, and 35.08%, respectively), TC/HDL-C (21.62%, and 26.45%, respectively), and LDL-C/HDL-C (19.48%, and 20.78%, respectively) in contrast to a significant reduction in the HDL-C (23.56%, and 29.84%, respectively) in the serum as compared with the control.

However, the FOD-Al_2_O_3_NPs and the FOD-ZnONP-treated groups recorded a significant reduction in all serum lipid parameters and their risks, except for HDL-C which recorded a significant elevation as compared with the Al_2_O_3_NPs, and ZnONP-administered groups, respectively.

In addition, the FOD-SiO_2_NP-treated group did not record any changes in all serum lipid parameters and their risks as compared with the SiO_2_NP-administered group.

### 3.7. The Effects of FOD on the Heart Histopathological Characters of Rats Administered with SiO_2_NPs, Al_2_O_3_NPs, or ZnONPs for 75 Days Are Represented in Figure 5

The control heart tissue showed a normal morphological appearance. However, the antioxidant-treated NP-administered groups (FOD-SiO_2_NPs, FOD-Al_2_O_3_NPs, and FOD-ZnONPs) recorded significantly ameliorated histopathological characters in the heart tissues as compared with the nonantioxidant-treated NP-administered groups (SiO_2_NPs, Al_2_O_3_NPs, and ZnONPs, respectively).

## 4. Discussion

Human and animal exposure to various nanoparticles increased with the progress in the field of nanotechnology increasing the possibility of detrimental health impacts due to exposure to these NPs [23, 36, 37]. As nanoparticles enter the body, the blood and lymph carry them to various human organs and tissues where they harm cells and cause significant cytotoxicities [38]. In agreement, the current study confirmed the blood and cardiovascular toxicity of subchronic exposure to SiO_2_NPs, Al_2_O_3_NPs, or ZnONPs due to induction of lipid peroxidation, oxidative stress, systemic inflammation, blood coagulation, endothelial dysfunction, myocardial enzymes, hematological parameter dysfunction, and many histopathological features [39, 40].

The nanoparticles can reach many organs via the circulatory system, so the NP's toxicity could be evaluated at the cardiovascular level [41]. The SiO_2_NPs, Al_2_O_3_NPs, and the ZnONP-administered groups for consecutive 75 days as subchronic oral administration recorded a significant reduction in the serum TAC, GSH, and SOD, in contrast to significant elevations in the serum ROS, and TBARS compared with the control as agreed by many previous studies [27, 40, 42] who recorded enhanced oxidative stress after exposure to different NPs through the generation of various ROS and RNS that interact with and cause damage to cells and finally causing cell death [27, 43]. The oxidative damage due to exposure to different NPs could alter the cell membrane permeability through the production of ROS that interacts with the phospholipids portion of the cell membrane and initiate the lipid peroxidation chain reaction and increases the production of TBARS [40, 44]. In agreement with the current data, different NPs were found to interact with different cell proteins, antioxidant defense mechanisms, ROS generation, and inflammatory response and eventually lead to apoptosis and necrosis [27, 40, 45].

Exposure to nanoparticles results in the activation of proinflammatory factors and proteins [46]. In agreement, the inflammatory status due to SiO_2_NPs, Al_2_O_3_NPs, or ZnONP administrations was confirmed by higher concentrations of the measured circulatory cytokines (TNF-*α*, IL-I*β*, and IL-6) which hint the inflammatory reaction [47, 48]. Different NP administrations could increase the inflammatory mediators and cause changes in the inflammatory cytokines [27, 49]. In line with the current study, amorphous silica (SiO_2_NPs) nanoparticles were found to significantly increase the inflammatory response and release of many cytokines [50]. Faddah et al. [51] found that ZnONP administration increases many inflammatory markers. Furthermore, Hou et al. [52] reported that entering Al_2_O_3_NPs into the systemic circulation stimulates the immune system and alters cytokine levels.

The diet of plants is important in the protection against free radicals attack and several diseases in the blood and cardiovascular due to the presence of many varied and potent natural antioxidants [53]. The current work confirmed that the used natural antioxidant (FOD) treatments for two weeks before and during the administration of the nanoparticles (75 days) effectively reduced oxidative stress and inflammatory markers induced by the NP administration. That could be due to the wide variety of antioxidants present in olive oil such as vitamin E, oleuropein, and carotenoids and to the cardiovascular, hepatoprotective, and anti-inflammatory effects of the oil. In addition, olive oil contains potent antioxidants that can scavenge free radicals and inhibit LDL oxidation [54]. It was recorded that the use of potent antioxidants to protect the lipids from the free radicals attack decreased lipid peroxidation significantly. Accordingly, olive oil phenolics were found to protect against LDL-C oxidation [55]. In agreement, the human diet containing olive oil for 3 weeks has led to decreased oxidized LDL-C together with increased TAC, GSH, and SOD activities [56]. In agreement with the current data, Saafi et al. [57] showed that the date fruit extract ameliorated the stress in the rats exposed to toxins as revealed by the inhibition of TBARS and enhancement of antioxidant parameters of TAC, SOD, CAT, and GSH. In agreement, the human supplementation with 40 g/day of dried figs was found to potently reduce the oxidative stress and the inflammatory markers [31].

The hematopoietic system is highly sensitive to the oxidative stress resulting from NP administration [42]. In agreement with the present study, the NP administration groups recorded significant alterations in various hematological parameters and blood indices that might be due to erythropoiesis failure, hemolysis of circulating cells, leakage of cells through the capillary wall, reduced cell production, or increased plasma volume [27, 50]. In addition, the hematological alterations in the present study caused by different NP administrations might be due to bone marrow syndrome [58]. The altered hematological values in the present study might be due to the damaged cell membranes from the increased lipid peroxidation products [59]. The administration of SiO_2_NPs, Al_2_O_3_NPs, or ZnONPs was associated with many hematological abnormalities as severe microcytic hypochromic anemia as agreed with previous studies [27, 50]. In agreement, the significantly elevated levels of WBC count in contrast to significantly reduced levels of RBC, Hb, HCT, and PLT in the NP-administered groups hinted the systemic inflammation [27, 50]. Following these results, Ben [60] and Yousef et al. [27] found that the administration of different NPs to rats caused anemia and many changes in the hematological parameters and a significant alteration in the cell indices (HCT and MCHC) which reinforces our findings.

The antioxidant-treated NP-administered groups showed significantly ameliorated hematological parameters as compared with the nonantioxidant-treated NP-administered groups. In agreement, the improvements in different hematological parameters in response to FOD supplementation for two weeks before and during the administration of the nanoparticles (75 days) might be attributed to the amelioration in the antioxidant enzymes. In addition, the improved Hct percentage might be attributed to improved liver functions [61, 62].

In agreement, Viola P. and Viola M. [63] attributed the improved hematological parameters against toxicity to the oleuropein and other active ingredients of the olive oil. In addition, Joseph and Raj [64] and Fathy [61] reported that figs are the ideal diet in anemic conditions. Moreover, Al-Jowari et al. [65] concluded that the aqueous fig extract improved the blood parameters and affected the hematopoiesis in the female rabbits. The figs are rich in fatty acids and vitamins which are necessary for the process of blood cell formation (hematopoiesis) in the red bone marrow and the production of the formed elements [66]. Interestingly, an active principle of ficin from this plant was shown to possess a hemostatic effect through the activation of factor X [67]. The present results also agree with Saafi et al. [57] who revealed the potent antioxidant phenolic acids in the date palm fruit extract. In the same concern, Wahab et al. [68] and Orabi and Shawky [69] confirmed the hemopoietic activity of the date palm fruits and the significant amelioration of the hematocrit, RBCs, WBCs, hemoglobin concentration, lymphocyte, monocyte, and neutrophil count after treatment of toxicity in rats with the date fruit extract.

As recorded in the present study, the cardiovascular toxicities of the SiO_2_NPs, Al_2_O_3_NPs, or ZnONPs show the effect of these NPs on blood circulation and the heart. In agreement, these toxicities could be attributed to the size of the NPs and their dosage schedule [27, 50]. In addition, different NPs can reach the heart via the cardiovascular system and can induce ROS production leading to cardiovascular dysfunction [41], so the NP toxicity could be proved at the level of the cardiovascular. In addition, increased amounts of ROS production and inflammatory response were involved in myocardial stunning, vascular dysfunction, and cell death [42, 70]. In line with the current data, the administration of silica, alumina, or zinc NPs to rats was found to enhance ROS production and cause endothelial inflammation and serious cardiovascular injury [40, 59].

The levels of sICAM-1 and sVCAM-l expression were elevated significantly in all NP-administered groups (SiO_2_NPs, Al_2_O_3_NPs, or ZnONPs) in the present study which reflect the endothelial cell injury and the tendency to thrombosis as agreeing with Du et al. [50]. In addition, these molecules can secret some agents which could affect the endothelial cells and aggravate WBCs adhesion to the vascular endothelium [71].

As a type-1 transmembrane protein, the cell adhesion molecule (CAM) P-selectin expression level increased in all NP-treated groups in the present study which reflect the coagulation system disorders through recruitment of leukocytes and platelets to the site of injury during inflammation [72]. Zhou et al. [7] and Du et al. [50] recorded that exposure to different NPs such as silica, alumina, or ZnO NPs could induce overexpression of P-selectin causing both DNA damage and cytokinesis block in blood vessels endothelial cells.

The potent vasoconstrictor endothelin-1 (ET-l) secreted from vascular endothelial cells significantly elevated in the SiO_2_NPs, Al_2_O_3_NPs, or ZnONPs administered to rats which may be attributed to atherosclerosis, endothelial injury, and heart failure [73, 74].

The small fibrin degradation product D-dimer is used as a marker for blood and coagulation disorders [75]. The increased D-dimer levels in the present study in all NPs administered groups could be attributed to the disseminated intravascular coagulation due to thrombosis and secondary fibrinolysis which is strongly related to cardiac events [50, 76]. The present results also suggest that the SiO_2_NPs, Al_2_O_3_NPs, or ZnONP administrations could activate the coagulation system due to thrombosis formation and could alter the stability of the coagulation/fibrinolytic system as agreed by many studies [50, 75].

The myocardial enzymes (CK, CK-MB, and LDH) are normally present inside the myocardial cells. The alteration in the plasma membrane integrity and/or permeability is reflected by the amount of these cellular enzymes in the blood [27, 77]. In agreement, the elevated serum myocardial enzymes in the present study in all NP-administered groups could be attributed to cell damage or ischemic necrosis and leakage of CK, CK-MB, and LDH into the circulation which are considered diagnostic markers of myocardial injury [42, 50].

The present study demonstrated that the antioxidant-treated NP-administered groups (FOD-SiO_2_NPs, FOD-Al_2_O_3_NPs, and FOD-ZnONPs) recorded significantly ameliorated blood coagulation, endothelial dysfunction, and myocardial enzymes parameters in the blood serum comparing with the nonantioxidant-treated NP-administered groups (SiO_2_NPs, Al_2_O_3_NPs, and ZnONPs, respectively). Defense against free radicals is fundamental to protect cellular molecules against several diseases including atherosclerosis and cardiovascular diseases [78, 79]. Therefore, there is a need to boost the antioxidant capacity and reduce releasing of free radicals [80]. Functional foods, such as olive oil, fig, and date palm, were good sources of exogenous antioxidants and are used in traditional medicine to manage various diseases [30, 81, 82]. Our results showed that FOD effectively ameliorated blood coagulation, endothelial dysfunction, and myocardial enzyme parameters induced by the NP administration indicating that FOD treatments have beneficial effects as antioxidants and protect against cardiovascular diseases [81, 82].

In agreement, the traditional Mediterranean diet including olive oil is beneficial against cardiovascular and atherosclerosis diseases due to its antioxidant activity, vasodilatory, antiplatelet aggregation, and anti-inflammatory effects [83, 84]. In the same concern, oxidation of the LDL-C was a hallmark of atherosclerosis and coronary heart disease. In addition, olive oil in the diet has been shown to reduce LDL-C oxidation [85].

The oxidative stress involved errors in lipid and protein metabolism that can induce liver injury and alterations in lipid metabolism [86]. All NP-administered groups in the current work recorded significant alterations in the lipids. In agreement, the SiO_2_NPs, Al_2_O_3_NPs, or ZnONPs administered to rats alter lipid metabolism and lipoproteins due to increased ROS production and hormonal imbalance [87]. This imbalance could induce hyperlipidemia from the adipose tissue's lipid mobilization or due to the decreased lipoprotein lipase activity that reduced the uptake of lipids by the adipose cells [88, 89]. Moreover, higher levels of cholesterol could result from the increased synthesis to restore the damaged membranes or from the increased activation of the HMG-CoA reductase enzyme. In agreement, the elevated serum triglyceride level may be attributed to the inhibition of enzyme activity leading to the reduction in the uptake of triacylglycerols and increasing the damage of cells due to lipid peroxidation [5, 70].

The alteration of lipid profile parameters after different NP administrations in the present study might be due to their interference with the lipid metabolism and decrease the cytochrome P450 which suppresses the cholesterol 7-hydroxylase that decreases the bile acids biosynthesis from cholesterol [42, 90]. NP administrations were also found to suppress the fatty acid *β*-oxidation and alter the lipids that might result in cardiomyopathy [91]. In agreement, the alteration of lipids in NP-treated groups might be due to the increased activity of cholesteryl esters synthetase and the inhibition of the carnitine palmitoyl-transferase system [92].

The present study demonstrated that the antioxidant-treated NP-administered groups (FOD-SiO_2_NPs, FOD-Al_2_O_3_NPs, and FOD-ZnONPs) recorded significantly ameliorated lipid profile compared with the nonantioxidant-treated NP-administered groups (SiO_2_NPs, Al_2_O_3_NPs, and ZnONPs, respectively). Our results showed that FOD effectively ameliorates lipid profile parameters induced by the NP administration. The increased ROS, LDL-C, triglycerides, and cholesterol are key factors for heart disease and may cause damage to the blood vessels. The ameliorative FOD results in the present study might be due to the olive oil's potent antioxidants that prevent lipid oxidation due to the prevention of cholesterol absorption or its production or increased cholesterol secretion and excretion [93, 94]. In agreement, unsaturated lipids like that of olive oil decreased the plasma cholesterol and ameliorated the blood lipids and the risk ratios [63]. In agreement with the present results, Lee et al. [95] recorded ameliorated lipid profile after fig extract supplementation to irradiated rats due to the protection of the plasma lipoproteins from oxidation and significantly elevated the plasma antioxidant capacity. In addition, the human supplementation with 40 g/day of dried figs potently reduced the oxidation of LDL-C [31, 96] and controls hyperlipidemia [97]. In agreement, the date palm fruit could cause activation of the hormone-sensitive lipase or lipogenic enzymes that caused decreased lipogenesis and increased lipolysis [98, 99]. Similarly, the date palm fruit inhibited LDL oxidation and stimulated the total cholesterol removal from the macrophages [100, 101].

In agreement, the heart tissue damage caused by SiO_2_NPs, Al_2_O_3_NPs, or ZnONPs is further confirmed by histopathological examination, which showed many pathological features including necrosis, scattered apoptotic (anucleated) cardiac muscle fibers, and others, showing small pyknotic nuclei with intracytoplasmic inclusions and markedly dilated thrombosed subpericardial and myocardial blood vessels with mild interstitial edema [27, 102].

The antioxidant-treated NP groups recorded significantly ameliorated histopathological characteristics compared with the nonantioxidant-treated NP groups. The protection of the heart tissues in response to FOD administration may be attributed to the decreased or prevented lipid peroxidation and protein oxidation due to the scavenging of the produced free radicals [27, 44].

## 5. Conclusion

The supplementation of FOD in NP-administered rats provides considerable protective effects against different NP-induced subchronic blood and cardiovascular toxicity in rats. The present findings also suggested the potential efficacy of FOD as a chemopreventive agent in the treatment of NP toxicity by modulating the oxidative stress, inflammatory markers, blood coagulation and endothelial dysfunction markers, myocardial enzymes, hematological parameters, lipid profile, and histopathological features.

## Figures and Tables

**Figure 2:**
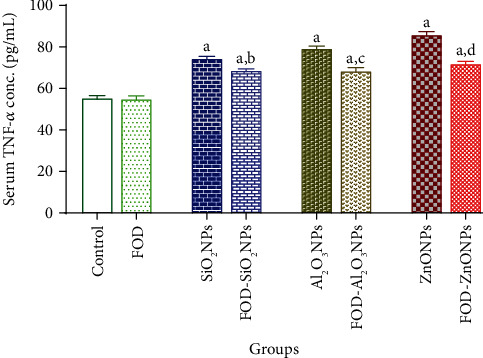
The effects of fig with olive oil and date-palm fruit extracts (FOD) on the serum tumor necrosis factor-*α* (TNF-*α*) in rats treated with different nanoparticles (NPs) for 75 days. ^a^*p* < 0.05 versus control group; ^b^*p* < 0.05 versus SiO_2_NPs; ^c^*p* < 0.05 versus Al_2_O_3_NPs; ^d^*p* < 0.05 versus ZnONPs. N.B: the antioxidants (FOD) treatments were used for 2 weeks before and during the administration of the nanoparticles.

**Figure 3:**
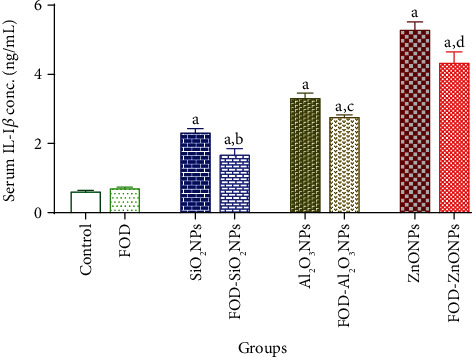
The effects of fig with olive oil and date-palm fruit extracts (FOD) on the serum interleukin-I*β* (IL-I*β*) in rats treated with different nanoparticles (NPs) for 75 days. ^a^*p* < 0.05 versus control group; ^b^*p* < 0.05 versus SiO_2_NPs; ^c^*p* < 0.05 versus Al_2_O_3_NPs; ^d^*p* < 0.05 versus ZnONPs. N.B: the antioxidants (FOD) treatments were used for 2 weeks before and during the administration of the nanoparticles.

**Figure 4:**
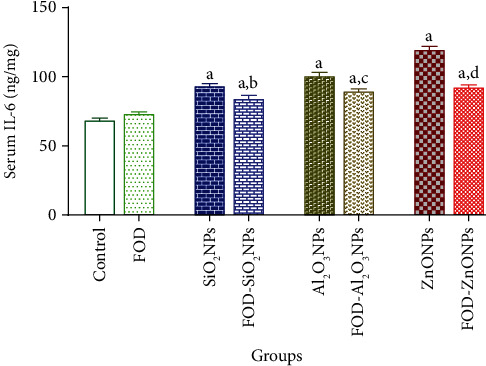
The effects of fig with olive oil and date-palm fruit extracts (FOD) on the serum interleukin-6 (IL-6) in rats treated with different nanoparticles (NPs) for 75 days. ^a^*p* < 0.05 versus control group; ^b^*p* < 0.05 versus SiO_2_NPs; ^c^*p* < 0.05 versus Al_2_O_3_NPs; ^d^*p* < 0.05 versus ZnONPs. N.B: the antioxidants (FOD) treatments were used for 2 weeks before and during the administration of the nanoparticles.

**Figure 5:**
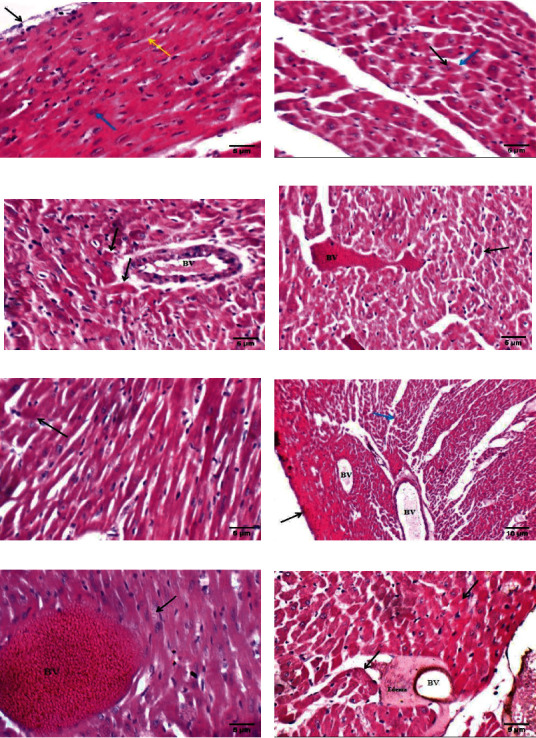
The effects of fig with olive oil and date palm fruit extracts (FOD) on the microscopic histological appearance of heart tissues treated with different nanoparticles (NPs) for 75 days. (a) High power view showing intact pericardium (black arrow), viable cardiac muscle fibers with central oval/elongated nuclei (blue arrow), and distinct cell borders (yellow arrow); (b) FOD-treated group showing viable cardiac muscle fibers with central elongated nuclei (black arrow) and distinct cell borders (blue arrow); (c) SiO_2_NP-treated group showing scattered apoptotic (anucleated) cardiac muscle fibers (black arrow) and average myocardial blood vessels (BV); (d) FOD-SiO_2_NP-treated group showing few scattered apoptotic (anucleated) cardiac muscle fibers (black arrow) and mildly dilated congested myocardial blood vessels (BV); (e) Al_2_O_3_ NP-treated group showing scattered apoptotic (anucleated) cardiac muscle fibers (black arrow); (f) FOD-Al_2_O_3_NP-treated group showing cardiac wall showing intact pericardium (black arrow), viable cardiac muscle fibers (blue arrow), and mildly dilated congested blood vessels (BV); (g) ZnO NP-treated group showing cardiac muscle fibers with scattered intracytoplasmic inclusions (black arrow) and markedly dilated and thrombosed myocardial blood vessels (BV); (h) FOD-ZnO NP-treated group showing few scattered apoptotic (anucleated) cardiac muscle fibers (black arrow) and mildly dilated blood vessels (BV) with mild perivascular edema (edema).

**Table 1:** The effects of fig with olive oil and date palm fruit extract on the serum oxidative stress markers of male rats treated with silicon oxide nanoparticles, aluminum oxide nanoparticles, or zinc oxide nanoparticles for 75 days.

Parameters	Experimental groups							
	Control	FOD	SiO_2_NPs	FOD-SiO_2_NPs	Al_2_O_3_NPs	FOD-Al_2_O_3_NPs	ZnONPs	FOD-ZnONPs
Serum TAC (mM/L)	1.87 ± 0.07	1.97 ± 0.01	1.18 ± 0.19^a^	1.46 ± 0.03^a,b^	0.88 ± 0.10^a^	1.17 ± 0.13^a,c^	0.77 ± 0.13^a^	1.11 ± 0.11^a,d^
		5.56%	-36.84%	-21.50%	-52.51%	-37.33%	-58.45%	-40.43%
Serum GSH content (mmol/L)	265 ± 1.10	262 ± 1.10	238 ± 6.07^a^	253 ± 2.81^a,b^	229 ± 6.16^a^	251 ± 1.81^a,c^	221 ± 5.40^a^	240 ± 1.67^a,d^
		-1.06%	-9.89%	-4.38%	-13.51%	-5.17%	-16.57%	-9.28%
Serum ROS conc. (ng/mL)	43.5 ± 1.57	41.2 ± 2.13	90.1 ± 2.60^a^	79.2 ± 3.08^a,b^	155 ± 9.31^a^	138 ± 7.73^a,c^	184 ± 2.68^a^	164 ± 3.72^a,d^
		-5.29%	107.13%	82.07%	257.93%	219.08%	325.06%	277.47%
Serum SOD activity (U/mL)	145 ± 1.05	140 ± 1.57	124 ± 1.42^a^	134 ± 2.17^a,b^	110 ± 3.00^a^	120 ± 3.56^a,c^	101 ± 2.31^a^	117 ± 2.26^a,d^
		-3.31%	-13.98%	-7.58%	-24.17%	-17.36%	-30.37%	-19.08%
Serum TBARS content (nmol/mL)	3.36 ± 0.11	3.19 ± 0.02	4.13 ± 0.13^a^	3.75 ± 0.07^a,b^	4.92 ± 0.23^a^	4.45 ± 0.23^a,c^	5.55 ± 0.18^a^	4.79 ± 0.13^a,d^
		-4.99%	22.87%	11.46%	46.39%	32.31%	64.86%	42.35%

Note: results are the mean ± standard error; %, percent of change from the control value; FOD: fig with olive oil and date-palm fruit extracts; SiO_2_NPs: silicon oxide nanoparticles; Al_2_O_3_NPs: aluminum oxide nanoparticles; ZnONPs: zinc oxide nanoparticles; TAC: total antioxidant capacity; GSH: reduced glutathione; ROS: reactive oxygen species; SOD: superoxide dismutase; TBARS: thiobarbituric acid reactive substances. For each parameter: ^a^*p* < 0.05 versus the control group; ^b^*p* < 0.05 versus SiO_2_NPs; ^c^*p* < 0.05 versus Al_2_O_3_NPs; ^d^*p* < 0.05 versus ZnONPs. N.B: the antioxidants (FOD) were used for 2 weeks before and during the administration of the nanoparticles (75 days).

**Table 2:** The effects of fig with olive oil and date palm fruit extract on the blood coagulation and endothelial dysfunction markers of male rats treated with silicon oxide nanoparticles, aluminum oxide nanoparticles, or zinc oxide nanoparticles for 75 days.

Parameters	Experimental groups							
	Control	FOD	SiO_2_NPs	FOD-SiO_2_NPs	Al_2_O_3_NPs	FOD-Al_2_O_3_NPs	ZnONPs	FOD-ZnONPs
Serum D-dimer conc. (ng/mL)	0.94 ± 0.01	1.00 ± 0.02	2.30 ± 0.12^a^	1.82 ± 0.11^a,b^	2.67 ± 0.14^a^	2.22 ± 0.07^a,c^	3.69 ± 0.12^a^	3.16 ± 0.19^a,d^
		6.55%	143.23%	93.34%	182.35%	134.78%	290.27%	234.78%
Serum ET-1 conc. (*μ*g/L)	48.1 ± 1.42	47.9 ± 1.50	81.6 ± 1.56^a^	64.7 ± 2.85^a,b^	123 ± 9.44^a^	107 ± 7.38^a,c^	177 ± 9.71^a^	152 ± 11.2^a,d^
		-0.42%	69.65%	34.51%	157.17%	123.70%	268.40%	217.46%
Serum P-selectin conc. (ng/mL)	2.15 ± 0.02	2.15 ± 0.06	4.22 ± 0.38^a^	3.15 ± 0.33^a,b^	4.11 ± 0.24^a^	3.27 ± 0.10^a,c^	4.83 ± 0.13^a^	4.01 ± 0.35^a,d^
		0.33%	96.56%	46.58%	91.31%	52.44%	124.73%	86.84%
Serum ICAM-1 conc. (ng/mL)	2.13 ± 0.01	2.23 ± 0.02	4.09 ± 0.24^a^	3.37 ± 0.09^a,b^	4.75 ± 0.15^a^	4.23 ± 0.18^a,c^	5.46 ± 0.34^a^	4.92 ± 0.17^a,d^
		4.35%	91.63%	57.81%	122.59%	98.04%	155.61%	130.54%
Serum VCAM-1 conc. (ng/mL)	1.82 ± 0.05	1.94 ± 0.07	3.98 ± 0.14^a^	3.5 ± 0.10^a,b^	4.42 ± 0.17^a^	4.04 ± 0.17^a,c^	4.97 ± 0.26^a^	4.42 ± 0.23^a,d^
		6.70%	118.83%	92.10%	142.97%	121.95%	172.89%	142.65%

Note: results are the mean ± standard error; %, percent of change from the control value; FOD: fig with olive oil and date-palm fruit extracts; SiO_2_NPs: silicon oxide nanoparticles; Al_2_O_3_NPs: aluminum oxide nanoparticles; ZnONPs: zinc oxide nanoparticles; ET-1: endothelin-1; ICAM-1: intercellular adhesion molecule-1; VCAM-1: vascular cell adhesion molecule-1. For each parameter: ^a^*p* < 0.05 versus the control group; ^b^*p* < 0.05 versus SiO_2_NPs; ^c^*p* < 0.05 versus Al_2_O_3_NPs; ^d^*p* < 0.05 versus ZnONPs. N.B: the antioxidants (FOD) were used for 2 weeks before and during the administration of the nanoparticles (75 days).

**Table 3:** The effects of fig with olive oil and date palm fruit extract on the myocardial enzymes and ALP of male rats treated with silicon oxide nanoparticles, aluminum oxide nanoparticles or zinc oxide nanoparticles for 75 days.

Parameters	Experimental groups							
	Control	FOD	SiO_2_NPs	FOD-SiO_2_NPs	Al_2_O_3_NPs	FOD-Al_2_O_3_NPs	ZnONPs	FOD-ZnONPs
Serum LDH activity (U/L)	529 ± 22.0	523 ± 14.0	812 ± 47.9^a^	687 ± 28.7^a,b^	984 ± 96.4^a^	820 ± 35.1^a,c^	991 ± 94.5^a^	849 ± 13.7^a,d^
		-1.11%	53.41%	29.82%	86.01%	55.01%	87.33%	60.51%
Serum CPK activity (U/L)	472 ± 15.5	469 ± 12.4	578 ± 26.9^a^	520 ± 18.5^a,b^	647 ± 12.3^a^	593 ± 16.5^a,c^	669 ± 19.7^a^	606 ± 15.8^a,d^
		-0.61%	22.43%	10.03%	36.90%	25.50%	41.62%	28.40%
Serum CK-MB (ng/mL)	0.12 ± 0.00	0.11 ± 0.00	2.42 ± 0.10^a^	1.74 ± 0.09^a,b^	4.91 ± 0.15^a^	3.75 ± 0.13^a,c^	5.27 ± 0.19^a^	4.21 ± 0.15^a,d^
		-8.59%	1793%	1265%	3736%	2830%	4017%	3194%
CK index	0.02 ± 0.00	0.02 ± 0.00	0.42 ± 0.02^a^	0.33 ± 0.02^a,b^	0.75 ± 0.02^a^	0.63 ± 0.03^a,c^	0.79 ± 0.04^a^	0.69 ± 0.02^a,d^
		-8.17%	1452%	1141%	2685%	2230%	2811%	2455%
Serum ALP (U/L)	185 ± 18.4	196 ± 13.7	254 ± 29.1^a^	203 ± 22.5^b^	307 ± 23.1^a^	241 ± 16.0^a,c^	318 ± 21.4^a^	264 ± 15.9^a,d^
		6.05%	37.31%	10.04%	66.04%	30.29%	71.92%	42.55%

Note: results are the mean ± standard error; %, percent of change from the control value; FOD: fig with olive oil and date-palm fruit extracts; SiO_2_NPs: silicon oxide nanoparticles; Al_2_O_3_NPs: aluminum oxide nanoparticles; ZnONPs: zinc oxide nanoparticles; LDH: lactate dehydrogenase; CK: creatine kinase; CPK: total creatine phosphokinase; CK-MB: MB isoenzyme of creatine kinase; ALP: alkaline phosphatase. For each parameter: ^a^*p* < 0.05 versus the control group; ^b^*p* < 0.05 versus SiO_2_NPs; ^c^*p* < 0.05 versus Al_2_O_3_NPs; ^d^*p* < 0.05 versus ZnONPs. N.B: the antioxidants (FOD) were used for 2 weeks before and during the administration of the nanoparticles (75 days).

**Table 4:** The effects of fig with olive oil and date palm fruit extract on the hematological parameters and blood indices of male rats treated with silicon oxide nanoparticles, aluminum oxide nanoparticles, or zinc oxide nanoparticles for 75 days.

Parameters	Experimental groups							
	Control	FOD	SiO_2_NPs	FOD-SiO_2_NPs	Al_2_O_3_NPs	FOD-Al_2_O_3_NPs	ZnONPs	FOD-ZnONPs
Hct (%)	44.6 ± 0.76	44.7 ± 0.80	35.8 ± 0.76^a^	38.5 ± 0.86^a,b^	30.3 ± 1.75^a^	33.8 ± 0.83^a,c^	27.7 ± 0.72^a^	30.1 ± 0.75^a,d^
		0.15%	-19.89%	-13.85%	-32.18%	-24.36%	-38.01%	-32.64%
RBC count (X10^6^/mm^3^)	6.11 ± 0.12	6.28 ± 0.11	5.43 ± 0.20^a^	5.86 ± 0.19^a,b^	4.72 ± 0.17^a^	5.44 ± 0.21^a,c^	4.42 ± 0.16^a^	4.96 ± 0.11^a,d^
		2.85%	-11.16%	-3.99%	-22.68%	-10.90%	-27.57%	-18.78%
Hb conc. (g/dL)	12.8 ± 0.35	12.8 ± 0.37	8.16 ± 0.19^a^	9.68 ± 0.34^a,b^	7.16 ± 0.15^a^	8.20 ± 0.26^a,c^	6.55 ± 0.27^a^	7.69 ± 0.42^a,d^
		-0.55%	-36.59%	-24.80%	-44.38%	-36.24%	-49.07%	-40.21%
MCV (fL)	73.5 ± 2.39	71.3 ± 1.94	66.4 ± 2.82^a^	66.1 ± 3.14^a^	64.6 ± 4.45^a^	62.5 ± 3.03^a^	62.9 ± 2.65^a^	60.8 ± 1.96^a^
		-2.94%	-9.59%	-10.07%	-12.03%	-14.84%	-14.34%	-17.28%
MCH (pg)	21.1 ± 0.81	20.3 ± 0.45	15.1 ± 0.73^a^	16.6 ± 1.00^a^	15.2 ± 0.71^a^	15.2 ± 0.87^a^	14.8 ± 0.59^a^	15.5 ± 0.80^a^
		-3.76%	-28.33%	-21.33%	-27.79%	-28.07%	-29.78%	-26.69%
MCHC (g/dL)	28.8 ± 0.68	28.6 ± 0.89	22.8 ± 0.82^a^	25.1 ± 0.82^a^	24.1 ± 1.75^a^	24.4 ± 1.18^a^	23.7 ± 1.02^a^	25.6 ± 1.62^a^
		-0.62%	-20.63%	-12.68%	-16.20%	-15.32%	-17.68%	-10.85%
Platelet count (10^3^/mm^3^)	712 ± 33.6	722 ± 25.1	531 ± 26.6^a^	614 ± 37.0^a,b^	475 ± 28.2^a^	599 ± 29.4^a,c^	486 ± 42.5^a^	574 ± 33.2^a,d^
		1.43%	-25.43%	-13.66%	-33.22%	-15.84%	-31.75%	-19.39%
WBC count (10^3^/mm^3^)	8.89 ± 0.68	8.51 ± 0.62	11.8 ± 0.20^a^	10.6 ± 0.40^a,b^	12.7 ± 0.35^a^	10.8 ± 0.50^a,c^	12.2 ± 0.38^a^	10.7 ± 0.56^a,d^
		-4.27%	33.60%	19.39%	43.70%	22.43%	37.64%	20.92%
Neutrophils percentage	19.3 ± 2.21	18.6 ± 1.72	30 ± 1.21^a^	24.4 ± 0.94^a,b^	31.2 ± 2.61^a^	25.6 ± 2.55^a,c^	33.7 ± 2.64^a^	25.3 ± 1.46^a,d^
		-3.42%	55.35%	26.35%	61.89%	32.57%	74.53%	31.01%
Lymphocytes percentage	53.1 ± 2.67	53.2 ± 2.47	65.5 ± 1.05^a^	59.9 ± 2.04^a,b^	70.7 ± 2.55^a^	59.7 ± 2.33^a,c^	77.0 ± 1.67^a^	60.7 ± 2.21^a,d^
		0.14%	23.18%	12.65%	32.96%	12.27%	44.93%	14.15%
Monocytes percentage	2.80 ± 0.29	2.87 ± 0.38	4.23 ± 0.18^a^	3.55 ± 0.16^a,b^	4.52 ± 0.33^a^	3.65 ± 0.20^a,c^	4.58 ± 0.48^a^	3.72 ± 0.16^a,d^
		2.32%	51.05%	26.63%	61.28%	30.12%	63.32%	32.66%

Note: results are the mean ± standard error; %, percent of change from the control value; FOD: fig with olive oil and date-palm fruit extracts; SiO_2_NPs: silicon oxide nanoparticles; Al_2_O_3_NPs: aluminum oxide nanoparticles; ZnONPs: zinc oxide nanoparticles; RBC: red blood corpuscle; Hb: hemoglobin; Hct: hematocrit; WBC: white blood cell count; MCV: mean corpuscular volume; MCH: mean corpuscular hemoglobin; MCHC: mean corpuscular hemoglobin concentration. For each parameter: ^a^*p* < 0.05 versus the control group; ^b^*p* < 0.05 versus SiO_2_NPs; ^c^*p* < 0.05 versus Al_2_O_3_NPs; ^d^*p* < 0.05 versus ZnONPs. N.B: the antioxidants (FOD) were used for 2 weeks before and during the administration of the nanoparticles (75 days).

**Table 5:** The effects of fig with olive oil and date palm fruit extract on the lipid profile and lipid risk ratios of male rats treated with silicon oxide nanoparticles, aluminum oxide nanoparticles, or zinc oxide nanoparticles for 75 days.

Parameters	Experimental groups							
	Control	FOD	SiO_2_NPs	FOD-SiO_2_NPs	Al_2_O_3_NPs	FOD-Al_2_O_3_NPs	ZnONPs	FOD-ZnONPs
Serum TG (mg/dL)	38.2 ± 2.94	37.7 ± 2.36	45.6 ± 0.75^a^	40.3 ± 2.45	56.9 ± 4.35^a^	48.6 ± 2.19^a,c^	59.2 ± 4.77^a^	51.6 ± 3.33^a,d^
		-1.31%	19.37%	5.50%	48.95%	27.23%	54.97%	35.08%
Serum TC (mg/dL)	51.8 ± 2.89	50.9 ± 1.17	60.3 ± 2.66*a*	56.5 ± 2.41	71.6 ± 3.89^a^	63 ± 2.54*a*^,c^	74.1 ± 4.62^a^	65.5 ± 3.53^a,d^
		-1.74%	16.41%	9.07%	38.22%	21.62%	43.05%	26.45%
Serum LDL-C (mg/dL)	23.1 ± 1.55	21.9 ± 1.25	27.9 ± 0.98^a^	25.1 ± 1.90	31.4 ± 1.65^a^	27.6 ± 1.72^a,c^	35 ± 1.66^a^	27.9 ± 1.59^a,d^
		-5.19%	20.78%	8.66%	35.93%	19.48%	51.52%	20.78%
Serum HDL-C (mg/dL)	19.1 ± 0.98	20.1 ± 1.80	15.6 ± 0.65^a^	17.8 ± 1.14	12.0 ± 0.61^a^	14.6 ± 0.75^a,c^	10.8 ± 0.61^a^	13.4 ± 0.55^a,d^
		5.24%	-17.84%	-6.68%	-37.04%	-23.56%	-43.32%	-29.84%
Serum non-HDL-C (mg/dL)	32.7 ± 3.19	30.8 ± 2.14	44.6 ± 2.72^a^	38.6 ± 2.53	59.5 ± 3.96^a^	48.4 ± 2.69^a,c^	63.2 ± 4.54^a^	52.1 ± 3.79^a,d^
		-5.81%	36.41%	18.27%	82.19%	48.01%	93.50%	59.33%
TG/HDL-C risk ratio (mg/dL)	2.24 ± 0.17	2.21 ± 0.13	2.68 ± 0.04^a^	2.37 ± 0.14	3.34 ± 0.25^a^	2.85 ± 0.12^a,c^	3.48 ± 0.28^a^	3.03 ± 0.19^a,d^
		-1.31%	19.37%	5.50%	48.95%	27.23%	54.97%	35.08%
TC/HDL-C risk ratio (mg/dL)	3.04 ± 0.17	2.99 ± 0.06	3.54 ± 0.15^a^	3.32 ± 0.14	4.21 ± 0.22^a^	3.70 ± 0.14^a,c^	4.35 ± 0.27^a^	3.85 ± 0.20^a,d^
		-1.74%	16.41%	9.07%	38.22%	21.62%	43.05%	26.45%
LDL-C/HDL-C risk ratio (mg/dL)	1.35 ± 0.09	1.28 ± 0.07	1.64 ± 0.05^a^	1.47 ± 0.11	1.84 ± 0.09^a^	1.62 ± 0.10^a,c^	2.05 ± 0.09^a^	1.64 ± 0.09^a,d^
		-5.19%	20.78%	8.66%	35.93%	19.48%	51.52%	20.78%

Note: results are the mean ± standard error; %, percent of change from the control value; FOD: fig with olive oil and date-palm fruit extracts; SiO_2_NPs: silicon oxide nanoparticles; Al_2_O_3_NPs: aluminum oxide nanoparticles; ZnONPs: zinc oxide nanoparticles; TG: triglycerides; TC: total cholesterol; HDL-C: high-density lipoprotein cholesterol; LDL-C: low-density lipoprotein cholesterol. For each parameter: ^a^*p* < 0.05 versus the control group; ^b^*p* < 0.05 versus SiO_2_NPs; ^c^*p* < 0.05 versus Al_2_O_3_NPs; ^d^*p* < 0.05 versus ZnONPs. N.B: the antioxidants (FOD) were used for 2 weeks before and during the administration of the nanoparticles (75 days).

## Data Availability

Data analyzed during this study are all included in the main manuscript.
